# Painless legs and moving toes syndrome associated with a sacral Tarlov cyst: a case report

**DOI:** 10.1186/s13256-016-0846-2

**Published:** 2016-03-09

**Authors:** Omar Alrawashdeh

**Affiliations:** Faculty of Medicine, Mutah University, Mutah Street, Mutah, 61710 Jordan

**Keywords:** Moving toes syndrome, Painless legs, Tarlov cyst

## Abstract

**Background:**

Painless legs and moving toes syndrome is a very rare syndrome characterized by continuous and involuntary movement of the toes. The etiology of the disease is not clear though it has been linked to a wide range of neuronal insults including proximal root compression and neuropathy. A previous study has reported bilateral painful legs and moving toes syndrome in a patient with a sacral Tarlov cyst. In this report we present a case of unilateral painless legs and moving toes syndrome in a woman with a sacral Tarlov cyst.

**Case presentation:**

A 50-year-old Mediterranean woman presented with a 1-year history of involuntary sustained movement of her right toes. Her physical examination and laboratory findings did not show any remarkable abnormality. Her lumbosacral magnetic resonance imaging scan showed a sacral Tarlov cyst. Our patient was given gabapentin, 100 mg per day as a starting dose, and showed modest improvement. Our patient preferred not to continue with the treatment as her symptoms were not disabling and she was only concerned about the cosmetic appearance.

**Conclusions:**

This report presents a new case of a very rare syndrome called painless legs and moving toes syndrome, which is possibly a variant of painful legs and moving toes syndrome. This is considered to be the first case of unilateral painless legs and moving toes syndrome that is associated with a sacral Tarlov cyst. Although the disease etiology is still unknown and the presence of the cyst can be accidental, neurologists should be aware that Tarlov cyst is a possible cause. In addition, patients with the painless variant who are not disabled by movement of the toes may not require treatment.

## Background

Painful legs and moving toes syndrome (PLMT) is a rare syndrome presenting with pain of the legs and involuntary movement of the toes and occasionally the feet. The first description of the syndrome was in 1971 by Spillane and colleagues [1]. They described six patients with painful legs and persistent involuntary movements of the toes [1]. Since then a number of reports have been published describing PLMT. Fewer reports have described a similar condition but without pain in the legs. The first report was published in 1993 and the condition was called painless legs and moving toes syndrome (PoLMT) [2].

The disease affects a wide age range as it has been reported in an 11-year-old girl and in an 86-year-old patient [3, 4]. There is no typical presentation of the disease but patients with PLMT syndrome usually present with bilateral leg pain and involuntary movements of the toes. Pain precedes movement of the affected limb by a few days to many years and it is described as a deep, irritating, burning or aching pain of the feet, ankles and legs.

The toe movement seems to have similar characteristics in PLMT and PoLMT. The toe movement has been described as sinuous, usually semirhythmic, a quivering, wriggling, writhing movement of the toes. Most of the time the movement is multidirectional and can be inhibited partially by voluntary and forceful contraction of the foot muscles [4, 5].

The etiology of PLMT and PoLMT is not clear, but it has been linked to a number of pathological conditions such as peripheral neuropathy, nerve trauma, or by root compression of the afferent fibers of the posterior roots ganglia such as cauda equina compression [4, 5].

In this report, we present a case of unilateral and painless movement of the toes in a woman who had a history of low back pain 15 years ago. A lumbar magnetic resonance imaging (MRI) scan showed a mild lumbar disc prolapse and a sacral Tarlov cyst.

## Case presentation

A 50-year-old Mediterranean woman presented with 1-year history of involuntary movement of the toes of her right foot. Our patient was not known to have diabetes or hypertension. Initially, the patient started to have an odd but painless feeling in her foot; she described the feeling as something moving inside her foot. This had gradually progressed to visible movement of the toes of her right foot; she did not describe any aggravating or relieving factors to the movement. However, the severity of movement varied during the day. There was no history of lower limb trauma or psychological problems. There was no history of neuroleptics use or symptoms of thyroid disease [5, 6]. Our patient reported that she had a history of low back pain 15 years ago. She had been told that surgery was required for her lower back pain but she did not recall the reason for the surgery; unfortunately, her previous MRI scan was not available.

On clinical examination, our patient appeared healthy with no signs of anxiety or psychological problems. She had a normal gait with normal tandem gait and a negative Romberg’s sign. The movement did not affect her gait and she could walk on her toes and her heels . Her upper and lower limb power and reflexes were normal. There was normal coordination of the upper and lower limbs with no evidence of cerebellar signs, nystagmus or ophthalmoplegia. Her peripheral pulses were intact but there was mild swelling of her feet and legs due to mild varicose veins.

The movement was a continuous semirhythmic movement involving the right first, second, third and fourth toes. It was a constant, flexion/relaxation movement with a variable frequency between 0.5 and 1 Hz. There was no associated visible movement of her ankle or calf muscles. The patient was able to temporarily suppress the movement by powerful extension of her toes and dorsiflexion of her ankle.

Laboratory investigations did not show any remarkable abnormalities. Her vitamin B12 level was normal and she was already on vitamin D3 treatment. A nerve conduction study of her right lower limb showed no evidence of demyelination or axonal loss. There was no neurophysiological evidence of peroneal nerve compression at the fibular head or tarsal tunnel syndrome. F wave examination and electromyography (EMG) did not show any evidence of denervation.

**Fig. 1:**
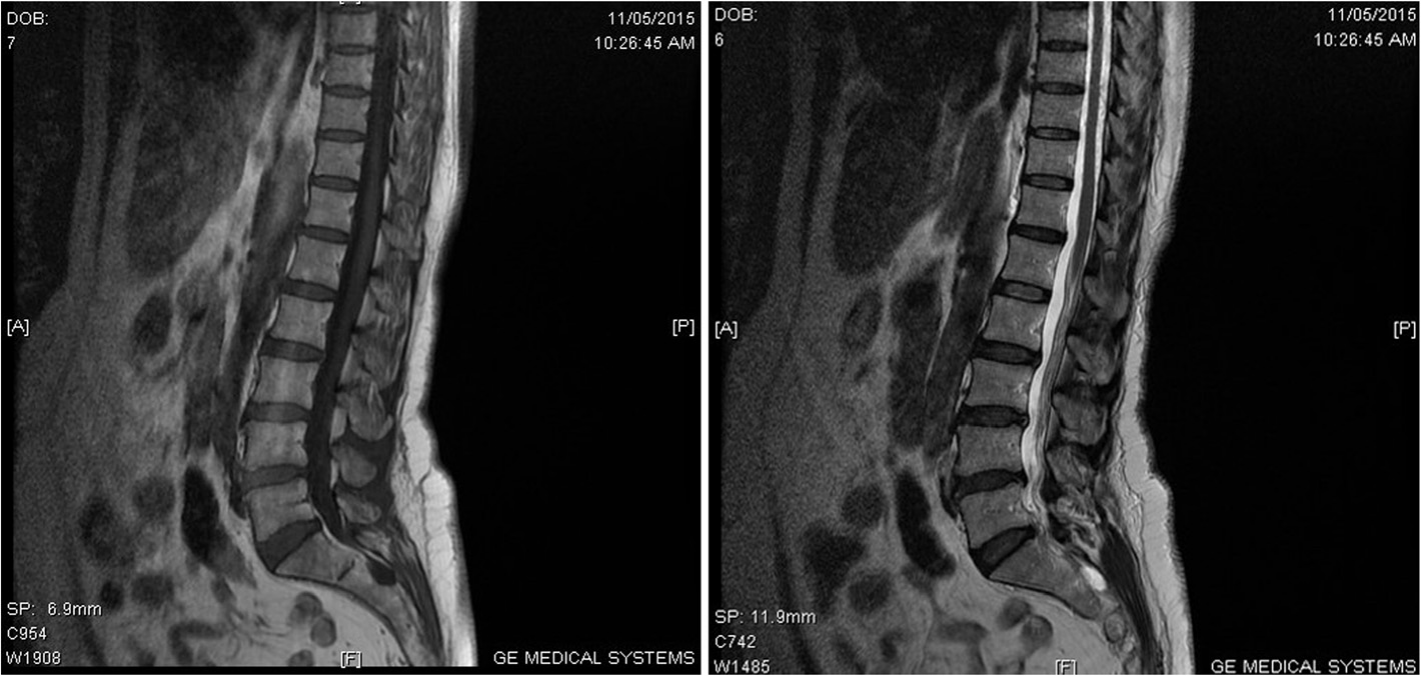
Lumbosacral magnetic resonance imaging scan showing a sacral Tarlov cyst (T1 and T2)

A lumbar MRI scan demonstrated a mild disc protrusion between L4 and L5. There was a much smaller disc protrusion between L5 and S1. Both discs did not show spinal cord or nerve root compression on axial view. A Tarlov cyst was seen at the sacral area (Fig. 1).

## Discussion

In this report, we presented a new case of PoLMT syndrome which is peculiar in that it is unilateral and a painless toe movement. The present case is probably the first report of PoLMT that is associated with a sacral Tarlov cyst. Only one previous report has linked sacral Tarlov cyst to bilateral PLMT syndrome [7]. PoLMT is much rarer than PLMT syndrome; until 2008, only six cases of PoLMT had been reported in the literature [8].

### Etiology

The etiology of PoLMT and PLMT remains unexplained and patients usually do not report a specific triggering factor for the symptoms and therefore many of the reported cases were considered idiopathic [3]. However, the two conditions have been linked to a number of neuronal insults including radiculopathy [9], traumatic nerve injury, and to peripheral nerve entrapment [10]. The most common reported risk factor for PLMT and PoLMT was disc compression or quada equina compression [5]. It is believed that root compression or peripheral nerve injury produce afferent signals that affect the dorsal horn and produce a central anomalous reorganization that frequently develops over many years [4, 5, 9]. This may explain why many patients with PLMT describe pain in the affected limb many years before the onset of movement. It is possible that the patient described in this report had a compression to the quada equina due to the presence of the Tarlov cyst. In fact, our patient had been experiencing abnormal but painless sensations in the affected foot for many years before the onset of the movement.

Such anomalous reorganization may act as a central oscillator that continuously produces signals. These signals probably reach the ventral horn neurons of certain myotomes and consequently produce rhythmic movement of the affected myotome (commonly the lower lumbar and upper sacral root myotomes where most of the disc prolapses take place).

However, the etiology may be more complicated than just a neuronal insult and the consequent central reorganization, PLMT and PoLMT have been associated with different pathologies in a number of previous reports. A reported case of PoLMT syndrome was linked to parasagittal meningioma [11]. Although most cases of PoLMT are sporadic, a genetic predisposition has been proposed as a risk factor for the disease. In 2003, Dziewas and his colleagues described bilateral PoLMT in a mother and her daughter without evidence of radiculopathy or neuropathy [12]. PoLMT has been described in a patient with Wilson disease and was temporary for 3 months and accompanied the extrapyramidal exacerbation of Wilson disease [5].

PLMT was also described to be temporary with the use of vincristine and metronidazole, symptoms subsided 6 weeks after stopping the treatment [13]. Neuroleptics and chemotherapy may also trigger the disease [5]. Other associated pathologies include Hashimoto’s thyroiditis [6], transverse myelitis [4], and herpes zoster myelitis [5]. Other possible associations of PLMT can be hormonal. PLMT is more common in females and symptoms may improve during pregnancy [14].

### Management

The disease can be primary or secondary. Cases which are associated with a certain pathology or related to drug intake are usually curable [5, 13]. Therefore, a thorough medical history and comprehensive clinical examination should be obtained to distinguish primary from secondary cases.

In PLMT syndrome, pain in the legs can be very severe and may interfere with gait. Pain may occasionally require opioid analgesia to be adequately controlled [4]. In a case series of 14 patients, the most effective treatment to relieve pain and movement was GABAergics including gabapentin, pregabalin and progabide [15]. Pain in the legs has been controlled with dual use of transcutaneous electrical nerve stimulation and vibratory stimulation [4].

In comparison, treatment of involuntary movement of the toes in PoLMT and PLMT syndromes has shown a modest success. A number of management strategies have been suggested including antiepileptics, antidepressants, benzodiazepines, local nerve block, sympathetic block, lumbar epidural block, and botulinum toxin type A injection. One case of PoLMT has shown complete control of toe movement with a low dose of clonazepam [16].

Different treatment strategies were discussed with the patient in this report including oral medication, nerve block, botulinum toxin, and surgical treatment of the cyst and the disc prolapse. It was explained that results obtained from these strategies were not guaranteed. Our patient was only concerned about the cosmetic appearance of her moving toes and thus preferred not to go through any invasive surgeries. An initial dose of gabapentin 100 mg once daily was prescribed. Our patient described a modest response to treatment. Finally, she preferred not to increase the dose and she voluntarily discontinued the treatment.

## Conclusions

This is probably the first reported case of unilateral PoLMT associated with a sacral Tarlov cyst. The previously reported case was associated with bilateral PLMT [7]. Although the presence of sacral Tarlov cyst may be accidental, neurologists should be aware that Tarlov cyst remains a possible cause. The case has also raised the question concerning the desired benefits from oral medication in patients who are not disabled by movement of the toes. Treatment of toe movement is usually required for a long period of time with no evidence of long-term cure. In addition, the disease course is classically not progressive and not fatal. PoLMT has been described in a 57-year-old man of 33 years’ duration without significant progression [2]. Therefore, avoiding medications in these patients may protect them from unnecessary side effects. However, follow-up of the patient’s condition and monitoring symptoms for any progression is recommended.

## Consent

Written informed consent was obtained from the patient for publication of this case report and any accompanying images. A copy of the written consent is available for review by the Editor-in-Chief of this journal.

## Abbreviations

EMG: Electromyography
MRI: Magnetic resonance imaging
PLMT: Painful legs and moving toes syndrome
PoLMT: Painless legs and moving toes syndrome
